# Prolonged Proton Pump Inhibitor Use and Electrolyte Abnormalities: A Case Presentation and Literature Review

**DOI:** 10.7759/cureus.87795

**Published:** 2025-07-12

**Authors:** Shadrack Ansong, Kaodi U Nwosu, Valentine Keke, Suganiya Rajoo, Moses Aboagye-Kumi

**Affiliations:** 1 Internal Medicine, Cape Fear Valley Medical Center, Fayetteville, USA; 2 Graduate Medical Education, Cape Fear Valley Medical Center, Fayetteville, USA; 3 Nephrology, Cape Fear Valley Medical Center, Fayetteville, USA

**Keywords:** drug-induced hypokalemia, duration of ppi use, electrolyte disturbances, hypernatremia, hypomagnesemia, ppi-induced, prolonged use of ppi

## Abstract

Proton pump inhibitors (PPIs) are utilized in the treatment of acid-related gastrointestinal diseases, such as gastroesophageal reflux disease (GERD) and peptic ulcers. Like any drug, they can cause side effects, some of which are observed during the clinical trial phase, while others become apparent during post-market surveillance. We present the case of a 68-year-old Caucasian female patient who had been taking a PPI (esomeprazole 40 mg twice daily) for over 10 years and presented to the Emergency Department (ED) with decreased magnesium levels (<0.5 mg/dL), hypokalemia (3.1 mmol/L), and hypocalcemia (<6.1 mg/dL), with no significant symptoms on initial presentation. The patient was treated with electrolyte repletion, and her medication was switched from omeprazole to famotidine upon stabilization of her electrolytes. She was discharged home with a follow-up appointment to further evaluate for channelopathies with Nephrology. The implications of this adverse effect underscore the importance of increased awareness among both healthcare providers and patients. Clinicians should consider the risk of PPI-induced hypomagnesemia, especially in patients with risk factors, and regularly monitor magnesium levels in individuals on long-term PPI therapy. Patients should be educated about the potential side effects of PPIs, including hypomagnesemia, and encouraged to report any concerning symptoms promptly.

## Introduction

Proton pump inhibitors (PPIs) are a class of medications designed to reduce the production of stomach acid. Some widely recognized examples of PPIs include omeprazole, lansoprazole, esomeprazole, pantoprazole, and rabeprazole [1,2]. These drugs are often prescribed to treat various medical conditions, such as gastroesophageal reflux disease (GERD), peptic ulcer disease (PUD), Zollinger-Ellison syndrome, infections caused by *Helicobacter pylori*, and to alleviate heartburn symptoms. Furthermore, PPIs help lessen the adverse effects that may arise from the use of other medications, particularly nonsteroidal anti-inflammatory drugs (NSAIDs) [1,3]. Due to their effectiveness, PPIs are among the most frequently prescribed medications and can also be purchased over the counter in some regions [3]. PPIs function primarily to affect parietal cells of the stomach. The parietal cells are responsible for producing hydrochloric acid in the stomach, which aids in the activation of enzymes needed for digestion and also deactivation of some viruses and bacteria in the stomach [1,4]. The hydrogen (H^+^)-potassium (K^+^) adenosine triphosphatase (ATPase) enzyme serves the purpose of producing acid in the stomach, which PPIs block [4]. PPIs are initially inactive and require a specific chemical transformation to become effective. This transformation occurs through an acid-catalyzed cleavage process that takes place within the acidic secretory canaliculi of parietal cells in the stomach [1,2]. Once activated, PPIs work to inhibit the proton pumps responsible for gastric acid secretion, thereby reducing acidity in the stomach.

These acid-reducing medications have gained widespread popularity due to their effectiveness in treating various gastrointestinal conditions and the initial belief that they are safe for long-term use. However, in recent years, studies and clinical observations have highlighted a concerning trend: the development of serious side effects linked to prolonged and high-dose consumption of these medications [3,5]. These adverse effects are not limited to one system but span multiple areas of health, including cardiovascular issues, gastrointestinal complications, bone density reduction, kidney problems, and disturbances in electrolyte balance. Some of the side effects of PPIs are *Clostridium difficile* infection, rebound acid secretion, and vitamin B12 deficiency. Vitamin B12 is bound to an R-factor, which needs to be cleaved to make the vitamin available for absorption. The proteases needed for such cleavage require an acidic medium for activation. Therefore, with PPIs, the acidic medium is not produced, which leads to malabsorption of the vitamin [1,3,5]. One of the most commonly reported side effects is hypomagnesemia, a condition characterized by low magnesium levels in the blood [6]. This deficiency can lead to a range of clinical manifestations, including muscle spasms, tetany (involuntary muscle contractions), irregular heart rhythms (arrhythmias), and even seizures, underscoring the importance of monitoring and managing the use of PPIs carefully. There are other reported side effects with limited data, including osteoporosis and calcium malabsorption [1]. 

This case report presents a detailed account of a patient who experienced hypomagnesemia due to the long-term use of PPIs, which also resulted in other electrolyte imbalances. Our primary aim is to highlight the potential complications associated with extended PPI therapy and to stress the importance of regularly monitoring magnesium levels in patients undergoing such treatment. Additionally, we delve into the underlying pathophysiological mechanisms that can contribute to the development of hypomagnesemia and examine how this condition can further lead to additional disturbances in electrolyte balance.

## Case presentation

A 68-year-old Caucasian female patient with a past medical history of hypertension, irritable bowel syndrime (IBS) with intermittent constipation and diarrhea, chronic lymphedema not on diuretics, hyperlipidemia, chronic GERD with gastritis on PPI, no alcohol use disorder, and fibromyalgia on pregabalin presented with multiple electrolyte abnormalities including hypokalemia, hypomagnesemia, hypocalcemia, and hypernatremia. The patient had basic laboratory tests done by her primary care physician (PCP) pending her upcoming annual primary care visit. The lab results showed magnesium levels of <0.5 mg/dL, calcium levels of 6 mg/dL, and potassium levels of 2.9 mmol/L. Her sodium levels were also elevated at 148 mmol/L. She was later called by her PCP, who instructed her to go to the nearest emergency facility due to derangements in her electrolytes.

On presentation to the Emergency Department (ED), the patient was in her normal state of health with no acute symptoms. She denied any shortness of breath, had no palpitations, no tetany, and no muscle cramps, but did admit to feeling fatigued for the past three to four days. The patient also denied any nausea, vomiting, or diarrhea and stated that her oral intake has been good with adequate intake of water. According to the patient, she was hospitalized about a month ago for electrolyte imbalance, at which time she was treated with intravenous (IV) magnesium and potassium and was discharged to rehab for two weeks due to generalized muscle weakness with no neurological deficits. During that admission, her magnesium levels were found to be in the range of 0.7 to 1.5 mg/dL while her potassium levels were in the range of 3.2 to 3.4 mmol/L. She had persistent nausea and vomiting and was seen by Gastroenterology, who increased her esomeprazole (Nexium) from 20 mg daily to 40 mg twice a day (BID) because of her history of gastritis. Because of her long-standing gastritis, she has been on PPI for more than a decade and had presented to the hospital some years ago for electrolyte imbalance and was asked to decrease her initial dosage of PPI from 40 mg BID to 20 mg daily. She was compliant with this until her last admission, when the dosage was again increased before this admission. 

In the ED, the patient was normotensive with a blood pressure of 117/77 mmHg, oxygen saturation of 98% on room air, temperature of 36.9°C (98.4°F), heart rate of 94 bpm, and respiratory rate of 18 breaths per minute. Initial labs, including a comprehensive metabolic panel (CMP) and complete blood count, are shown in Tables 1-2. Thyroid-stimulating hormone (TSH) was normal. Magnesium levels were found to be very low (Table 3). 

**Table 1:** Comprehensive metabolic panel in the Emergency Department (H): High; (L): Low; BUN: Blood urea nitrogen; eGFR: Estimated glomerular filtration rate; AST: Aspartate aminotransferase; ALT: Alanine transaminase

Component (Reference Range)	Value
Sodium (136-145 mmol/L)	149 (H)
Potassium (3.4-4.9 mmol/L)	3.1 (L)
Chloride (98-107 mmol/L)	106
CO_2_ (21-32 mmol/L)	31
BUN (7-25 mg/dL)	7
Creatinine (0.60-1.30 mg/dL)	0.51 (L)
Glucose (74-109 mg/dL)	83
Calcium (8.6-10.2 mg/dL)	6.2 (L)
AST (13-39 U/L)	17
ALT (7-52 U/L)	7
Alkaline phosphatase (30-105 U/L)	71
Total protein (6.4-8.9 g/dL)	6.1 (L)
Albumin (3.5-5.7 g/dL)	3.4 (L)
Bilirubin total (0.3-1.0 mg/dL)	0.5
eGFR (>60.0 mL/min/1.73 m^2^)	>60.0
Anion gap (1-11 mmol/L)	10

**Table 2:** Complete blood count in the Emergency Department (L): Low; (H): High; MCV: Mean corpuscular volume; MCH: Mean corpuscular hemoglobin; MCHC: Mean corpuscular hemoglobin concentration; MPV: Mean platelet volume; RDW-CV: Red cell distribution width-coefficient of variation

Component (Reference Range)	Value
WBC (4.5-12.5 x10^3^/uL)	6.5
RBC (4.20-5.40 x10^6^/uL)	3.91 (L)
Hemoglobin (12.0-16.0 g/dL)	11.0 (L)
Hematocrit (36.0-48.0%)	33.9 (L)
MCV (81.0-99.0 fL)	86.6
MCH (27.0-31.0 pg)	28.1
MCHC (31.0-36.0 g/dL)	32.5
Platelets (150-450 x10^3^/uL)	271
MPV (7.4-10.4 fL)	7.4
RDW-CV (11.7-14.4%)	17.2 (H)

**Table 3:** Magnesium levels in the Emergency Department (LL): Low panic

Component (Reference Range)	Value
Magnesium (1.9-2.7 mg/dL)	<0.5 (LL)

**Figure 1:**
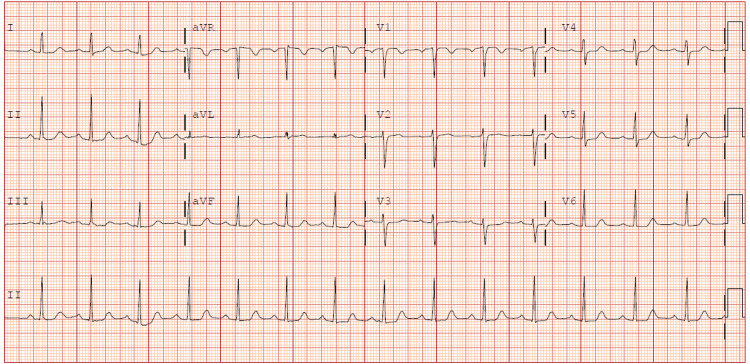
Initial ECG at time of presentation to the Emergency Department with normal sinus rhythm and no T-wave changes

An electrocardiogram done in the ED showed normal sinus with no T-wave changes (Figure 1). 

The patient received 2 g of IV magnesium, calcium gluconate 1 g IV, and potassium chloride (KCl) 20 mEq IV in the ED and was admitted for further management. The patient was started on lactated ringers after her free water deficit was calculated to be 2.3 L. Her magnesium and potassium levels were still low, with magnesium being below 1.2 mg/dL and potassium at 3.2 mmol/L. Her calcium levels were 6.4 mg/dL with normal albumin. Another repletion was done with 2 g of IV magnesium, 40 mEq of potassium, and 2 g of calcium gluconate. Urine electrolytes were obtained, and Nephrology was consulted due to long-standing hypomagnesemia. Upon further chart review, it was noted that the patient was seen and evaluated by Cardiology about six years ago, where she was started on diltiazem (Cardizem) 120 mg daily after presenting with premature ventricular complexes (PVCs) on ECG. The patient stated that this medication was started after she was given a Zio® Patch (cardiac event monitor) (iRhythm Technologies, USA) for three weeks and diagnosed with PVCs around the same time she started presenting with electrolyte abnormalities, particularly hypomagnesemia. Cardiac event monitor readings were not available for review, but notes from her cardiologist mentioned a PVC burden of more than 25%. 

We obtained her urine electrolytes, and PPI was stopped after review by Nephrology. Urine electrolytes are shown in Table 4. 

**Table 4:** Urine electrolyte levels during her hospital stay

Component (Normal Range)	Value
Urinary sodium (<20 mmol/L)	165
Urinary chloride (110-250 mmol/L)	91
Urinary potassium (25-125 mmol/L)	12.8

Table 5 depicts the trend of her magnesium levels until discharge.

**Table 5:** Magnesium levels throughout hospitalization (LL): Low panic; (L): Low

Component (Reference Range)	Day 1	Day 2	Day 3	Day 4
Magnesium (1.9-2.7 mg/dL)	<0.5 (LL)	1.9 (evening after repletion)	2.6	2.2
Magnesium (1.9-2.7 mg/dL)	<0.5 (LL)	0.9 (LL) (morning before repletion)	1.8 (L)	2.3

After stopping PPI, her magnesium levels stabilized along with her potassium and calcium levels; her sodium levels improved after 24 hours of lactated ringers at 100 mL/hr. She was discharged on a histamine-2 (H2) blocker (famotidine 20 mg twice daily) for her gastritis. The patient was scheduled to follow up with Nephrology for further evaluation to rule out any channelopathies that might exist outside the effect of PPI. Two months after stopping PPI, she was seen by her nephrologist, who did some basic labs, including CMP, parathyroid hormone (PTH) levels, and urine electrolytes. The results are listed in Table 6.

**Table 6:** Labs obtained at post-hospitalization follow-up with Nephrology BUN: Blood urea nitrogen; eGFR: Estimated glomerular filtration rate; PTH: Parathyroid hormone

Component (Reference Range)	Value
Glucose (70-100 mg/dL)	85
BUN (7-20 mg/dL)	9
Creatinine (0.74-1.35 mg/dL)	0.63
eGFR (>60.0 mL/min/1.73 m^2^)	97
BUN/creatinine ratio (10-20)	14
Sodium (136-145 mmol/L)	144
Potassium (3.4-4.9 mmol/L)	3.8
Chloride (98-107 mmol/L)	105
Bicarbonate (CO_2_) (21-32 mmol/L)	28
Calcium (8.6-10.2 mg/dL)	10
Phosphorus (2.5-4.5 mg/dL)	3.2
Albumin (3.4-5.4 g/dL)	3.8
Magnesium (1.7-2.2 mg/dL)	1.9
PTH (10-65 pg/mL)	51
Urinary magnesium (24 hr) (50-200 mg/24 hr)	<30
Urinary calcium (24 hr) (100-300 mg/24 hr)	136

## Discussion

PPIs are a type of medication that permanently inhibit the H^+^-K^+^ ATPase enzyme, also known as the proton pump, in the stomach’s parietal cells. These drugs start as prodrugs that require an acidic environment of the stomach for activation. Once activated, they bind covalently to cysteine residues on the H^+^-K^+^ ATPase enzyme, resulting in long-lasting suppression of acid production until new proton pumps are generated [6]. Due to this mechanism, PPIs are more effective than H2 receptor antagonists in raising gastric pH and aiding the healing of acid-related tissue damage [5,6].

Many countries have made PPIs available without prescription as they are generally regarded as safe; however, studies have documented several side effects. Common mild reactions include headaches, abdominal pain, nausea, vomiting, diarrhea, constipation, and flatulence [2,3,7]. More severe complications are linked to prolonged use, such as a higher risk of bone fractures, *Clostridium difficile*-associated diarrhea, low magnesium levels and other electrolyte imbalances, vitamin B12 deficiency, chronic kidney disease, and pneumonia.

Our case report describes a 68-year-old Caucasian female patient with a history of PVCs, GERD, and gastritis who had been on long-term PPI since 2006. She had presented multiple times with severe hypomagnesemia (≤0.5 mg/dL) while being on PPI for her gastritis. During her previous visit to the hospital before this current admission, she exhibited several electrolyte imbalances; however, these were initially attributed to a three-day episode of nausea and vomiting. As a result, her PPI was temporarily discontinued but restarted upon discharge. Approximately a month later, she returned with similar electrolyte disturbances, prompting the indefinite discontinuation of her PPI.

Magnesium deficiency is a rare but potentially serious side effect associated with this class of medications, first identified in a 2006 case report [2]. Since then, multiple case reports and studies have confirmed this link [2,8,9]. In 2011, the Food and Drug Administration (FDA) issued a warning about the risk of hypomagnesemia, particularly with prolonged PPI use [3,8]. The agency recommended monitoring serum magnesium levels in patients on long-term PPI therapy, especially those also taking other medications like diuretics that can further lower magnesium levels [3,7].

There are two methods by which magnesium ions (Mg^2+^) are taken up in the intestine from the body. These include passive diffusion across enterocytes and active transport facilitated by channels [9]. The process of passive magnesium absorption exhibits characteristics such as nonlinearity, low affinity, and a dependence on the concentration of magnesium present on the luminal aspect of the gut. About 7% of ingested magnesium is absorbed through this straightforward diffusion method, which implies that higher luminal concentrations correlate with increased absorption rates using this method of absorption [5,9]. The active transport of magnesium in the intestinal tract is mediated by the collaborative roles of transient receptor potential melastatin 6 and 7 (TRPM6/7) channels that are found in the apical membrane of enterocytes. These high-affinity channels are saturated when magnesium levels in the gut are high, and when low, the channels play a vital role in magnesium absorption due to the high affinity of the channels for Mg^2+^, allowing the body to adapt to lower levels of dietary intake [6-8].

Most studies indicate that in people experiencing hypomagnesemia related to PPI use, the kidneys appropriately reduce magnesium excretion, which is normally seen with urinary magnesium levels of <30 mg/L and a decrease in calcium and potassium levels in the serum [9]. One of the postulated mechanisms by which PPI decreases magnesium levels is by decreasing the active absorption of magnesium in the small intestine. PPIs raise the pH in the intestinal lumen, reducing magnesium solubility and hindering its absorption [9]. Another theory suggests that PPIs interfere with magnesium transporters, such as TRPM6/7, which are crucial for active magnesium uptake in the intestines. This impairment can be more pronounced in individuals with certain genetic mutations affecting TRPM6, leading to more severe magnesium deficiency over time [9].

Hypomagnesemia is a condition marked by unusually low magnesium levels in the blood, typically defined as serum magnesium below 0.7 mmol/L (1.7 mg/dL) [3]. Magnesium is an essential electrolyte that plays a key role in various physiological functions, including muscle and nerve activity, blood sugar regulation, and bone health [10]. PPI-induced hypomagnesemia can lead to a range of clinical symptoms affecting multiple organ systems. The most common effects involve neuromuscular, cardiovascular, and metabolic disturbances, which may present as muscle cramps, tremors, tetany, seizures, and cardiac complications such as ventricular arrhythmias and sudden cardiac death [2,3]. Metabolic imbalances related to hypomagnesemia include hypocalcemia and hypokalemia. Hypocalcemia arises from reduced PTH secretion and function, while hypokalemia occurs due to increased potassium loss through the kidneys and decreased absorption in the enterocytes [8,9]. Magnesium is vital for the proper function of the sodium (Na^+^)-potassium (K^+^) ATPase channel used for potassium absorption in the enterocytes. A deficiency in magnesium significantly decreases the function of this channel, leading to hypokalemia. Another method by which hypokalemia occurs is through the renal outer medullary potassium (ROMK) channel responsible for potassium regulation in the kidneys. A decrease in intracellular magnesium, caused by magnesium deficiency, releases the magnesium-mediated inhibition of ROMK channels and increases potassium secretion in the collecting tubule of the nephron [10]. When this happens, potassium repletion is often ineffective until magnesium levels are corrected. Magnesium also regulates the sodium (Na^+^)-chloride (Cl^-^) cotransporter (NCC), and its deficiency reduces NCC abundance via neuronal precursor cell downregulation 4-2 (NEDD4-2), preventing its activation during hypokalemia, which results in increased distal sodium delivery, further promoting potassium loss and worsening hypokalemia [10].

Hypocalcemia can also result from chronic PPI-induced hypomagnesemia. A deficiency in magnesium can inhibit the secretion of PTH. In cases of severe hypomagnesemia, PTH release is paradoxically blocked because low magnesium levels can mimic the activation of the calcium-sensing receptor (CASR) in the parathyroid gland, suppressing hormone secretion [3]. Additionally, magnesium deficiency reduces the kidneys’ responsiveness to PTH, decreasing calcium reabsorption and increasing calcium loss in urine (calciuria), which contributes to hypocalcemia [10]. Beyond renal effects, low magnesium levels can also lead to skeletal resistance to PTH, meaning that even when PTH is produced, the skeletal system does not respond properly, impairing calcium release into the bloodstream [3].

To treat PPI-induced hypomagnesemia, the first step is discontinuing the PPI, if possible, as this usually resolves the issue. Magnesium should be replaced orally or intravenously, depending on the patient’s condition and deficiency severity, along with other electrolytes. If stopping the PPI is not feasible, on-demand or intermittent PPI therapy may be a better option [8,10]. Alternatively, H2 receptor antagonists and other antacids can be used instead. Overall, the American Gastroenterological Association emphasizes the need for routine monitoring of magnesium and electrolytes in patients on long-term PPI therapy, especially elderly individuals and those taking medications that may exacerbate the imbalance [8]. In this case presentation, to rule out other possible causes of the hypomagnesemia, the patient was given a two-month interval for laboratory testing including urinary electrolytes and serum potassium, magnesium, and calcium levels, which showed improvement of all electrolytes after stopping the PPI without the patient being on any magnesium, calcium, or potassium supplements. 

## Conclusions

This case highlights the importance of carefully evaluating patients presenting with electrolyte disturbances, particularly focusing on magnesium, potassium, and calcium levels, as imbalances in these electrolytes often occur concurrently. The patient in question experienced hypomagnesemia while taking a PPI. In such instances, discontinuing the PPI and switching to an alternative class of antacids, such as H2 blockers, may be a crucial step in mitigating the side effects. However, it is imperative to conduct a thorough differential diagnosis, ruling out other potential causes of electrolyte imbalance. Conditions such as renal dysfunction, malnutrition, and various medical treatments can also lead to similar clinical presentations. By meticulously analyzing all relevant factors, clinicians can effectively determine whether the PPI is indeed responsible for the electrolyte abnormalities or if another underlying cause exists. This case emphasizes the critical role of vigilant monitoring and proactive management in patients taking PPIs to ensure optimal patient outcomes and prevent serious complications associated with electrolyte imbalances.
